# EEG Correlates of Self-Managed Neurofeedback Treatment of Central Neuropathic Pain in Chronic Spinal Cord Injury

**DOI:** 10.3389/fnins.2019.00762

**Published:** 2019-07-25

**Authors:** Aleksandra Vučković, Manaf Kadum Hussein Altaleb, Matthew Fraser, Ciarán McGeady, Mariel Purcell

**Affiliations:** ^1^Rehabilitation and Assistive Devices, Biomedical Engineering Division, School of Engineering, University of Glasgow, Glasgow, United Kingdom; ^2^Faculty of Electrical Engineering, Wasit University, Wasit, Iraq; ^3^Queen Elizabeth National Spinal Injuries Unit, Queen Elizabeth University Hospital, Glasgow, United Kingdom

**Keywords:** central neuropathic pain, spinal cord injury, electroencephalography, neurofeedback, sensory-motor rhythm

## Abstract

**Background:**

Neurofeedback (NFB) is a neuromodulatory technique that enables voluntary modulation of brain activity in order to treat neurological condition, such as central neuropathic pain (CNP). A distinctive feature of this technique is that it actively involves participants in the therapy. In this feasibility study, we present results of participant self-managed NFB treatment of CNP.

**Methods:**

Fifteen chronic spinal cord injured (SCI) participants (13M, 2F), with chronic CNP equal or greater than 4 on the Visual Numeric Scale, took part in the study. After initial training in hospital (up to 4 sessions), they practiced NF at home, on average 2–3 times a week, over a period of several weeks (min 4, max 20). The NFB protocol consisted of upregulating the alpha (9–12 Hz) and downregulating the theta (4–8 Hz) and the higher beta band (20–30 Hz) power from electrode location C4, for 30 min. The output measures were pain before and after NFB, EEG before and during NFB and pain questionnaires. We analyzed EEG results and show NFB strategies based on the Power Spectrum Density of each single participant.

**Results:**

Twelve participants achieved statistically significant reduction in pain and in eight participants this reduction was clinically significant (larger than 30%). The most successfully regulated frequency band during NFB was alpha. However, most participants upregulated their individual alpha band, that had an average dominant frequency at α_p_ = 7.6 ± 0.8 Hz (median 8 Hz) that is lower than the average of the general population, which is around 10 Hz. Ten out of fifteen participants significantly upregulated their individual alpha power (α_p_ ± 2 Hz) as compared to 4 participants who upregulated the power in the fixed alpha band (8–12 Hz). Eight out of the twelve participants who achieved a significant reduction of pain, significantly upregulated their individual alpha band power. There was a significantly larger increase in alpha power (*p* < 0.0001) and decrease of theta power (*p* < 0.04) in participant specific rather than in fixed frequency bands.

**Conclusion:**

Neurofeedback is a neuromodulatory technique that gives participants control over their pain and can be self-administered at home. Regulation of individual frequency band was related to a significant reduction in pain.

## Introduction

Neuropathic pain is a chronic condition caused by damage to or disease of the somatosensory nervous system (Haanpää et al., 2011), affecting 7–10% of the general population (Colloca et al., 2017). Although the cause of pain is in the nervous system, it is typically perceived as burning or stinging sensation coming from the body (Siddall et al., 2003). Neuropathic pain may be of a peripheral (i.e., post operative, cancer, and painful diabetic neuropathy) or of a central origin (Seifert and Maihöfner, 2009). Central neuropathic pain (CNP) is caused by an injury to the central nervous system including the brain and the spinal cord, and it has a high prevalence in conditions such a spinal cord injury (SCI) (Siddall et al., 2003; Finnerup, 2013), multiple sclerosis (Osterberg et al., 2005), stroke (Andersen et al., 1995) and Parkinson’s disease (Beiske et al., 2009).

In people with SCI, the prevalence of neuropathic pain is around 50% and it typically occurs within the first year of injury (Siddall et al., 2003). Severe CNP results in reduced quality of life (Middleton et al., 2007) due to its impact on sleep and anxiety (Siddall et al., 2003; Finnerup, 2013) and in more extreme cases also affects employability (Mann et al., 2013).

There are six main mechanisms involved in the chronification of neuropathic pain: (i) activity increase in areas of the pain neuromatrix, (ii) recruitment of additional cortical areas beyond the classical pain neuromatrix, (iii) cortical reorganization and maladaptive neuroplasticity, (iv) alterations in neurochemistry, (v) structural brain changes, and (vi) disruption of the brain default mode network (Seifert and Maihöfner, 2009).

Central neuropathic pain is typically treated with medications, such as antidepressant, anticonvulsants and opioids (Dickenson and Ghandehari, 2007). These medications have multiple side effects, such as drowsiness, nausea, constipation and dry mouth (Bakonja and Rowbotham, 2006) and some of them lead to misuse or even drug-related deaths (Gov.UK, 2019). In addition to having side effects, medications have a limited efficacy (Finnerup et al., 2018). For example, about half the people taking gabapentin experience a moderate reduction in pain (30%) (Wiffen et al., 2017).

Neuromodulatory treatment of CNP typically rely on external electrical or magnetic stimulation such as repetitive Transcranial Magnetic Stimulation (rTMS), transcranial Direct Current Stimulation (tDCS), and Cranial Electrotherapy Stimulation (CES) (O’Connell et al., 2018). A recent Cohrane review showed that rTMS on average results in 12%, while tDCS results in 17% short term relief in pain. There was no evidence of CES effectiveness. O’Connell et al. (2018) suggested 15% as a clinically relevant reduction in pain, meaning that only tDCS may result in a clinically significant reduction in pain. Typically, tCDS electrodes are applied over the motor cortex while rTMS is applied either over the pre-frontal or central area. It is believed that while both techniques target the central motor area, they indirectly influence cortical areas involved in the pain matrix. For this reason, stimulation sites do not necessarily correspond to the somatotopic location of the part of the body that is perceived as being painful.

Neurofeedback is a neuromodulatory intervention which does not require an external electrical or magnetic filed. It is a type of a biofeedback where users are provided with real time information about their brain activity in a form of a visual, audio or even haptic feedback. Neurofeedback allows users to develop a voluntary control over an unconscious physiological process using a feedback signal (Sherlin et al., 2011; Ros et al., 2014). Thus it enables the implicit control of covert brain activity that may have no direct behavioral correlates.

Neurofeedback has been used for decades to treat various disorders, most successfully attention deficit hyperactivity disorder and epilepsy (Demos, 2005). Development of inexpensive Brain Computer Interface technology contributed to its recent popularity not only for the treatment of neurological conditions but also for the improvement of peak performance in the able bodied people (Gruzelier, 2014). NFB has been used for treatments of various types of pain including complex regional pain syndrome (Jensen et al., 2007) fibromyalgia (Kayiran et al., 2010; Caro and Winter, 2011), trigemina neuralgia (Sime, 2004), cancer pain (Hetkamp et al., 2019), migraine (Siniatchkin et al., 2000), and CNP (Jensen et al., 2013a; Hassan et al., 2015).

The advantage of NFB over other neurostimulation techniques is that it enables the participants to take the active part in therapy, changing the locus of control from the external to the internal (Wood and Kober, 2018). People who use NFB for a prolonged period of time can learn to apply the technique at will without a feedback (Sherlin et al., 2011). Furthermore the technique is less costly than rTMS and is also potentially safer than rTMS, tDCS, and CES. From a research perspective, NFB has an additional advantage over neurostimulation techniques, it records brain activity thus providing direct evidence of neuromodulation during the therapy.

Neurofeedback is often based on operant conditioning, a strategy that increases a preferred behavior and decreases an undesired behavior providing a reward or punishment (Skinner, 1948). The idea behind this is to train a user to promote a desirable response to occur again under the same conditions (Sherlin et al., 2011). Typically in NFB a “reward” and a “penalty” are presented in a visual form, e.g., changes in the color or size of an object on a computer screen. Simple, frequently used forms of reward and penalty are changes in color (i.e., reward corresponds to green color while penalty corresponds to red color) or size of on object on a computer screen.

It is believed that NFB tunes brain oscillations toward a homeostatic set-point which affords an optimal balance between network flexibility and stability (i.e., self-organizing criticality) (Ros et al., 2014). It is believed that non-degenerative brain disorders may have a self-tuning impairment, having their dynamic repertoire “trapped” in an abnormal resting state (Ros et al., 2014). In this respect, chronic pain disrupts “a default mode network” (Baliki et al., 2008). NFB facilitates global brain connectivity and establishes a normalized default mode network.

In order to establish a NFB protocol for CNP it was necessary to identify how CNP affects brain oscillatory activity, as measured by EEG. An important factor influencing oscillatory EEG activity is thalamo-cortical dysrhythmia, that serves as a trigger for cortical dysfunction (Llinas et al., 1999; Walton and Llinas, 2010). Thalamo-cortical dysrhythmia is caused by either deafferentation or disinhibition of thalamic nuclei and as such may have a subcortical or supracortical origin such as neuropathic pain, tinnitus, Parkinson’s disease and depression (Llinas et al., 1999). In case of CNP caused by SCI, a lesion to the spinal cord causes deafferentation of thalamic nuclei leading to hyperpolarization of these cells. When hyperpolarized, thalamic neurons change from high threshold tonic firing to low threshold theta range oscillatory burst. Such low frequency oscillations entrain corticothalamic loops generating increased coherence between the thalamus and the cortex, and increased power in 4–8 Hz range in the cortical level, also accompanies by the reduction of the dominant (alpha) frequency (Llinas et al., 1999). At the same time thalamocortical modules in theta mode exert less collateral inhibition to the neighboring modules, which are thereby activated in higher beta and gamma frequency ranges (Llinas et al., 1999; Sarnthein et al., 2006). This abnormal high frequency firing is proposed to generate the positive symptoms of pain and allodynia (Schulman et al., 2005). In people with CNP, increased theta band activity is considered the main signature of CNP (Sarnthein et al., 2006; Stern et al., 2006; Boord et al., 2008; Vuckovic et al., 2014).

Previous EEG studies on CNP in SCI also reported a shift in the dominant alpha frequency toward lower values and a reduced reactivity (reduction of EEG power) to eyes opening, attributed to the altered input from the thalamus (Boord et al., 2008; Jensen et al., 2013b; Vuckovic et al., 2014). Changes in the resting state alpha band power are reported in some studies to be increased (Vuckovic et al., 2014) while others reported decreased alpha band power compared with participants with no pain (Jensen et al., 2013b). Our group also found an increased level of desynchronisation during imagined movements of both painful and non-painful limbs (Vuckovic et al., 2014), supporting results from fMRI studies indicating the over-activity of the sensory-motor cortex due to CNP (Wrigley et al., 2009; Gustin et al., 2010).

A recent review of EEG patterns in chronic pain found that the increased theta and alpha activity at rest and decreased amplitude of evoked potentials to sensory stimulation and cognitive tasks are the main indices of chronic pain (Pinheiro et al., 2016). On the contrary, Camfferman et al. (2017) suggested that the reduced alpha band power is an indicator of chronic pain in general. A discrepancy with respect to alpha band power in different studies may be partially explained by the way alpha band power was calculated, as in people with CNP peak activity is lower. Thus standard 8–12 Hz band might not reflect the individual alpha band power.

In our recent study we showed that a reduced alpha power and a reduced dominant alpha frequency are predictive asymptomatic markers of CNP in people with SCI (Vuckovic et al., 2018b). These markers appear before pain and are representative features for machine learning systems that can predict the risk of developing pain (Vuckovic et al., 2018a). This indicates that alpha activity may be the most relevant feature for a NFB protocol.

The NFB protocols for treatment of different types of chronic pain show that most protocols target the central or temporal cortex, upregulating alpha (8–12 Hz) or lower beta (12–15 Hz) activity and downregulating theta (4–8 Hz) and higher beta (20–30 Hz) (Kayiran et al., 2010; Caro and Winter, 2011; Jensen et al., 2013a; Hassan et al., 2015). All protocols were based on fixed frequency bands.

In our previous study we tested protocols that increased the alpha (9–12 Hz) and decreased the theta and higher beta band over the primary motor cortex (C3 and C4) (Hassan et al., 2015). Four out of five participants who received 20 to 40 sessions reported a more than 30% reduction in pain. The effect was notable up to 1 month following the last session. We also demonstrated that all participants were able to modulate their brain activity in the direction of NFB even without the feedback. On the contrary, with pre-recorded sham feedback provided they were not able to modulate their brain activity. Finally we showed that a week following the last session, their event related desynchronisation over the sensory-motor cortex during imagined movement was reduced, indicating reduced over activity of the motor cortex (Hasan et al., 2016). Their baseline EEG was also reduced in a wider pain matrix including the dorsolateral prefrontal cortex, the anterior cingulate cortex and the insular cortex, in the higher beta band.

In this participant self-managed study, we asked participants with long standing CNP and SCI to practice NFB on their own and monitored their pain level and EEG activity. The advantage of self-managed NFB is that users can decide when to use the NFB, thus potentially maximizing the effect of treatment.

Research questions that we are trying to answer in this paper are:

• Can people learn how to self-manage NFB for CNP?
• Are NFB bands for treatment of CNP fixed or patient specific?
• What is the relation between self-regulated brain activity and the experience of pain?

## Materials and Methods

### Participant Demographics

Twenty people with SCI (4 female and 16 male, aged 50.6 ± 14.1 years), previously diagnosed with CNP (Mahnig et al., 2016) participated in this study. The American Spinal Injury Association (ASIA) Impairment Classification was used to determine the neurological level of SCI (Kirshblum et al., 2011), and the completeness of injury. The level of injury C (cervical) corresponds to tetraplegia while T (thoracic), and L (lumbar) to paraplegia. The completeness of injury is defined as: A-sensory and motor complete, B-sensory incomplete and motor complete and C and D-sensory and motor incomplete. Eight participants were able to walk, nine were fulltime wheelchair users who had good hand function and three participants were tetraplegic with poor hand function. Tetraplegics were assisted by their caregivers with NFB setup.

There were no inclusion restrictions with respect to the level or completeness of the injury, as there is no clear evidence of correlation between these factors and the incidence of CNP (Siddall et al., 2003). Table 1 shows participants’ demographic information. The inclusion criteria were: intensity of CNP ≥ 4 on a Visual Numerical Scale (VNS, 0 = no pain, 10 = worst pain imaginable), CNP ongoing for at least 6 months (chronic), aged between 18 and 75 years, no self-reported history of brain disease or injury, and basic computer skills. The exclusion criteria were: presence of chronic or acute muscular or visceral pain ≥ 4 VNS, traumatic brain injury, stroke, epilepsy, or any other self-reported neurological problem.

**TABLE 1:** Participants’ demographic information.

**Nr**	**ASIA level**	**ASIA comp**	**Pain (VNS)**	**Yeas since injury**	**Type of CNP**	**Med**
P1	L3/L4	D	9	10	Un	P
P2	T6/T7	D	7	7	Un	G
P3	T5	D	3	7	At/Un	T
P4	T4	A	7	6	Un	T
P5	L3	D	5	5	Un	/
P6	C2	B	5	8	Un	P
P7	C2	A	7	5	Un	/
P8	C3/C5	D	3	5	At/Un	/
P9	T5	A	7	5	Un	P
P10	C4	D	15	10	Un	G
P11	C2	A	1	4–5	Un	G
P12	T6	B	1	5	At/Un	D
P13	T5	D	10	6	Un	/
P14	C6/C7	A	5	6	Un	/
P15	T6/T7	A	30	9–10	un	N
P16	L3/4	D	7	4	Un	/
P17	C5-C7	A	15	6	Un	B/P
P18	L4	A	21	6	Un	B/P
P19	L2	A	13	7	Un	/
P20	T10	A	1	8	Un	B

*Nr, participant’s number; ASIA, Americal Spinal Injury Association; VNS, Visual Numerical Scale, CNP, Central Neuropathic Pain; Med, medications; P, pregabaline; G, gabapentin; T, tramadol; D, duloxetine; N, nabline.*

**FIGURE 1:**
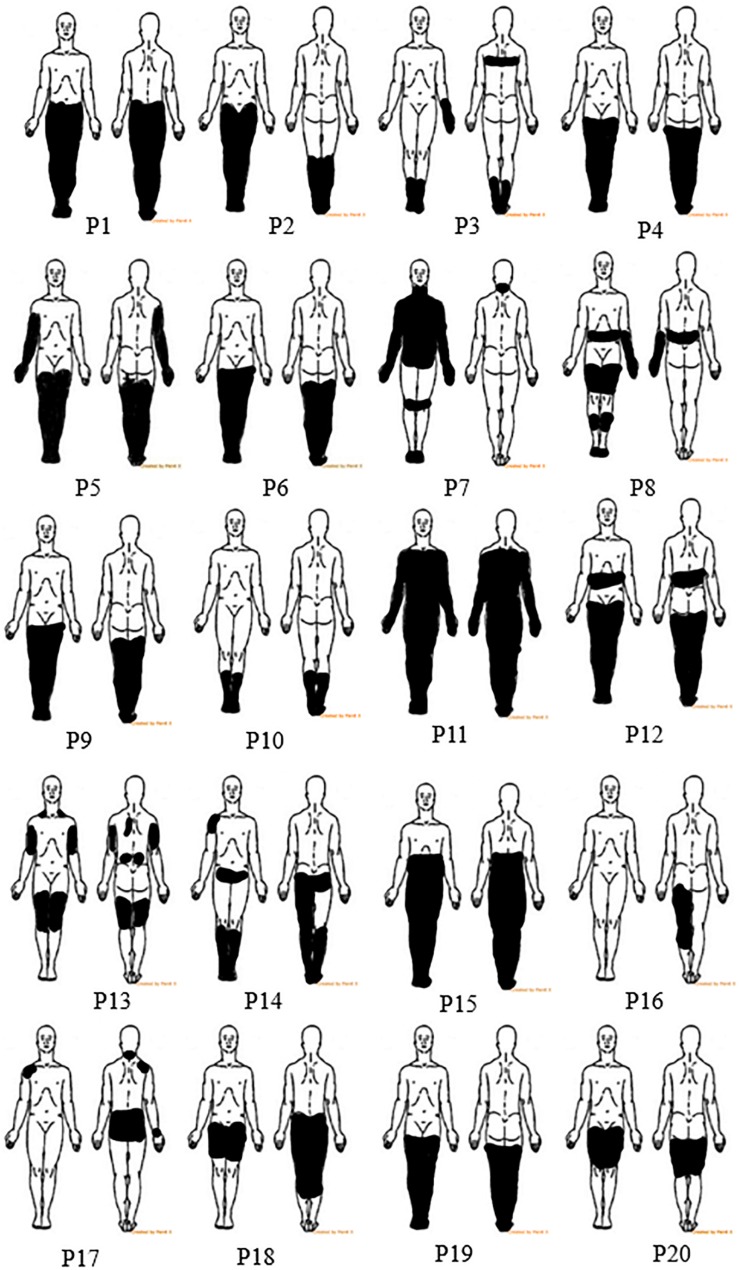
Body charts showing pain location for all 20 participants.

All participants had below level pain while participants 3, 8, and 12 also had pain at the level of injury. The location of the painful region was marked by participants on a body chart (Figure 1). At level pain may occur due to injury to the roots or spinal cord, thus it may have central or peripheral origin. Below level pain is more common and more persistent; it has a central origin and is caused by the injury to the spinal cord (Bryce et al., 2012; Finnerup et al., 2014). Participants typically described the pain sensation as pins and needles, constant burning or freezing, tingling or squeezing combined with the intermittent electrical shock sensations.

**FIGURE 2:**
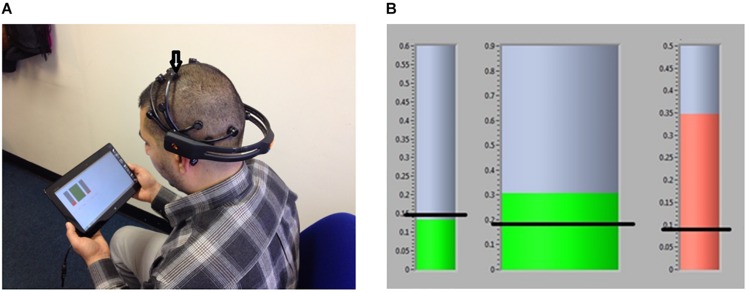
**(A)** A member of the research team (who consented that his photo is provided) demonstrate the correct location of the EEG headset with an arrow pointing to the electrodes from which the EEG was provided. **(B)** Neurofeedback GUI; Horizontal black lines present an example of threshold values, they were not shown to users.

Most of the participants were prescribed CNP medications, such as antidepressants or anticonvulsants. They were asked not to change their medications throughout the study to avoid their interference with the NFB effect on pain. Six patients previously tried non-pharmacological treatments (acupuncture and mindfulness) that were available through the National Healthcare Service, but have not been receiving any non-pharmacological treatment for at least 6 months prior to taking part in the study. During a regular check-up communication, approximately once a fortnight (in person, email, or Skype) there were asked if there were any changes dosage or type of medication. All patient participants and able-bodied participant shown in Figure 2 provided written informed consent. The study has been approved by the Greater Glasgow and Clyde National Health Service Ethical Committee. This study is a registered clinical trial NCT02678494.

### The Experimental Protocol

#### Initial Assessment

At the beginning of the study, all participants were asked to fill out a “Brief Pain Inventory” (Tan et al., 2004) and a “Neuropathic Pain Symptom Inventory” (Bouhassira et al., 2004). Participant’s initial response to NFB was measured using a laboratory device usbamp (Guger Technology, Austria), as in our previous study (Hassan et al., 2015; Hasan et al., 2016). This devices uses an EEG cap and can therefore precisely define the electrode locations. This is how the location of electrodes for the wearable EEG headset (Epoch, Emotiv, United States) was determined. Based on the literature (Jensen et al., 2013a) and our initial assessment of NFB on able-bodied people (Al-Taleb, 2018), we organized up to four initial assessment sessions in hospital. We were looking for early sensory responses to NFB such as: reduction in pain of at least one point on the VNS, a pleasant warmth replacing the sensation of burning or freezing, tingling in the toes or finger tips (Hassan et al., 2015). Five out of twenty participants decided to withdraw before completing all four NFB sessions, three could not commit to the study and two were lacking any response to NFB.

#### Participant Training to Use a Wearable BCI

The remaining fifteen participants and caregivers were trained to use the Epoch EEG (Emotiv, United States) device and custom made software. They were offered up to 4 training sessions before taking the EEG device and tablet with software home to train on their own. To minimize the number of hospital visits, some of the training sessions took place on the same day as the assessment with the usbamp. Participants were instructed to practice the NFB on demand but at least once a week for a period of 2 months; some participants decided to keep the system after 2 months, we followed them up till the end of the study. All participants were also asked to fill out an electronic pain diary before and after training. The intensity of pain was recorded using the VNS ranging from 0 (no pain) to 10 (worst pain imaginable) in integer values. EEG was recorded during NFB sessions from the training electrode. At the end of the training, semi-structured interviews were organized. The results of the interviews with participants will be analyzed in a usability study presented elsewhere.

Because Epoch was not initially designed to record from the central cortex, participants were trained to place the headset on the correct central location, so that the recording electrode was placed approximately in between C2 and C4. The Epoch headset is only available in one size it was therefore not possible to provide a more precise location. Figure 2A shows an example of correct placing of the headset with location for the electrode from which training was provided.

#### System Description and Neurofeedback Training Protocol

NFB training was provided approximately from the electrode location C4 (American Clinical Neurophysiology Society [ACNS], 2006), placed over the primary motor cortex of the left arm and hand. The sampling frequency was 128 samples/s and two reference electrodes were placed parietaly, above the ears for CMS/DRL noise cancelation. The impedance was set under 10 kΩ using Epoch proprietary software. A wireless communication between the EEG device and tablet, was based on proprietary 2.4 GB wireless technology.

Custom made software written in C++ (Al-Taleb, 2018) consisted of a unit for EEG recording and analysis and a Graphical User Interface (GUI). The GUI had the main screen for neurofeedback training, a screen for EEG parameters setting, and an electronic pain diary.

Neurofeedback sessions always started with 2 min baseline recording. During the baseline recording, participants sat still with their eyes open looking at the center of the screen. NFB training sessions were 5 min long and participants were advised to have 5–6 daily sessions.

A training protocol was adopted from the previous study by our group (Hassan et al., 2015). During NFB training the power of the EEG signal was calculated over a 0.5 s sliding window. EEG signal was filtered in four frequency bands: wideband 2–30 Hz, Θ theta (4–8 Hz), α alpha (9–12 Hz), and higher β beta (20–30 Hz) using a 5th order Butterworth filter. The relative power was calculated as a power in a selected band divided by wide band 2–30 Hz power. Relative power was presented to users during NFB (range from 0 to 1, corresponding to 0 to 100% of the wide band power).

The values of relative theta, alpha and beta power were shown on a computer screen, in a form of three separate bars (Figure 2B). During NFB training, participants were aiming at increasing the alpha band power and decreasing the theta and beta band power, relative to the baseline power recorded on the same day prior to NFB. A slightly higher alpha range starting from 9 rather than 8 Hz was selected in order to shift the dominant frequency toward the higher values, as a lower dominant alpha frequency was found to be a signature of CNP (Boord et al., 2008). While the theta and beta bands were related to EEG signatures of pain, these two bands are also related to noise coming from blinking (theta band) and muscle activity (beta band). Thus minimizing theta and beta power also minimized the online noise.

The training threshold was fixed and set to 110% of the baseline alpha power and to 90% of the baseline theta and higher beta power. A color of the representative bar was green when the alpha power was above the threshold and was red otherwise. For the theta and beta power the color of the representative bars was green when the power was under the threshold and red otherwise. The bars also changed size proportional to the amplitude of the power.

#### Off-Line Analysis

##### Removal of artifact

EEG was visually inspected and signal having an amplitude greater than 100 μV or containing electrooculogram EOG (blinking and eye rolling) was manually removed. On average 10–20% of the signal was removed.

##### Power spectrum density

PSD was calculated using Hamming windows over 4 s long recording overlapping for 2 s and averaging PSD over all windows (Welch periodogram method). A logarithmic PSD was calculated as 10*log*_10_⁡*PSD* for visualization purposes.

Power was calculated for (4–8 Hz), (8–12 Hz), and (20–30 Hz) and for individual frequency bands by summing up PSD values over the selected frequency range. In order to determine the individual bands, the frequency of alpha peak α_p_ was determined first as a maximum of PSD graph and a band power was calculated with respect to that peak. The individual alpha band was defined as (α_*p*_± 2 Hz), the individual theta band as (α_p_−6 Hz to α_p_−2 Hz) and the individual higher beta band as (α_p_+12 Hz to α_p_+22 Hz).

##### Statistical analysis

Mann–Whitney *U*-test was used to compare VNS pain intensity before and after neurofeedback and EEG data. A non-parametric Spearman correlation coefficient was calculated between the intensity of pain and EEG features as well as between pain and patient demographic data. A significance level of *p* = 0.05 was adopted in all cases.

## Results

### Participants Compliance With the Study

Out of fifteen participants who took part in the NFB study, seven used the system for 2 months as required. Three out of these seven continued to use the system for additional 6 weeks. Eight participants discontinued the study for the following reasons: unrelated health problems (*N* = 3), new caregiver (*N* = 1), moving home (*N* = 1), too long donning time (>15 min) (*N* = 2), broken device (*N* = 1). Out of seven participants who used the system in their home for 8 weeks or longer, five could walk, one was a sensory and motor complete paraplegic, and one sensory and motor complete tetraplegic. EEG data and pain diaries were collected from all fifteen participants (participants could not start NFB or exit the software application unless filling out the pain diary). Table 2 shows the number of session, frequency of use and the total duration of the study for each participant. Participants were advised to use NFB on demand, when they had time and when they felt most pain. From the frequency of use, which in most participants was about three times per week, it can be concluded that NFB had an effect that lasted several days. This is, however, only an estimate, because participants did not fill out the pain diary on a daily basis and at the same time of the day (participants often reported that pain tends to be higher in the evening than in the morning).

**TABLE 2:** The number of NFB sessions.

**Part. code**	**Nr sessions**	**Total Nr subsess.**	**Weekly usage**	**Duration (weeks)**
P1	3	12	1–2	2
P2	12	30	1	8
P3	7	24	3–5	2
P4	40	235	3–6	12
P5	48	280	3–6	14
P6	\	\	\	\
P7	9	31	3	3
P8	\	\	\	\
P9	\	\	\	\
P10	14	84	1–3	8
P11	3	16	1–3	4
P12	6	29	1–3	3
P13	\	\	\	\
P14	10	42	3–4	3
P15	\	\	\	\
P16	20	108	3–5	8
P17	31	143	3–5	9
P18	4	21	1–3	3
P19	3	12	1	3
P20	3	17	1–2	2

### The Ability to Regulate EEG Power

**FIGURE 3:**
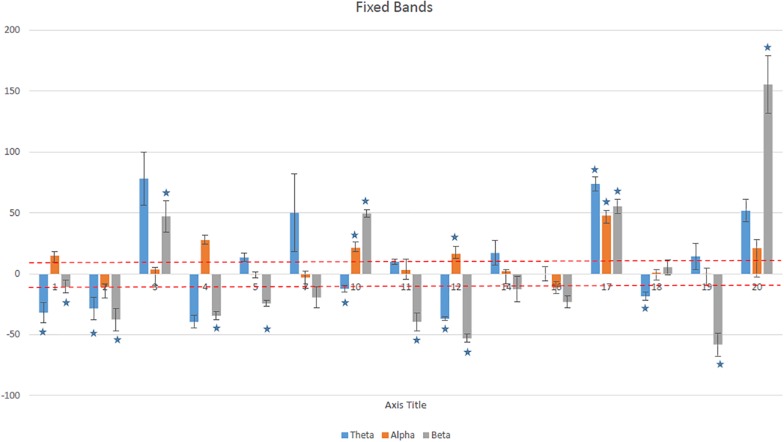
The average percentage of change (mean ± SD) over all training sessions for each participant in theta (4–8 Hz), alpha (8–12 Hz), and higher beta (20–30 Hz) bands. Asterisks show statistically significant values.

We analyzed the average ability of each participant to upregulate the alpha and to down regulate the theta and beta band power. Figure 3 shows the average (mean ± SD) modulation of power, with respect to the baseline power in fixed frequency ranges (theta, alpha, and higher beta) for each participant. Positive values correspond to the upregulation (increase) while negative values correspond to the downregulation (decrease). A dashed line represents 10% change with respect to the baseline ( ± 10% was also set as a training threshold). In addition, a statistical analysis was performed over all training sessions, to assess whether NFB consistently modulated EEG power in a desired direction (increase of the alpha and decrease for the theta and beta bands). This was a somewhat conservative approach as it also included early sessions while participants were still learning the NFB technique. Only three participants significantly upregulated their alpha band power, but nine participants significantly downregulated either theta or beta band power.

**FIGURE 4:**
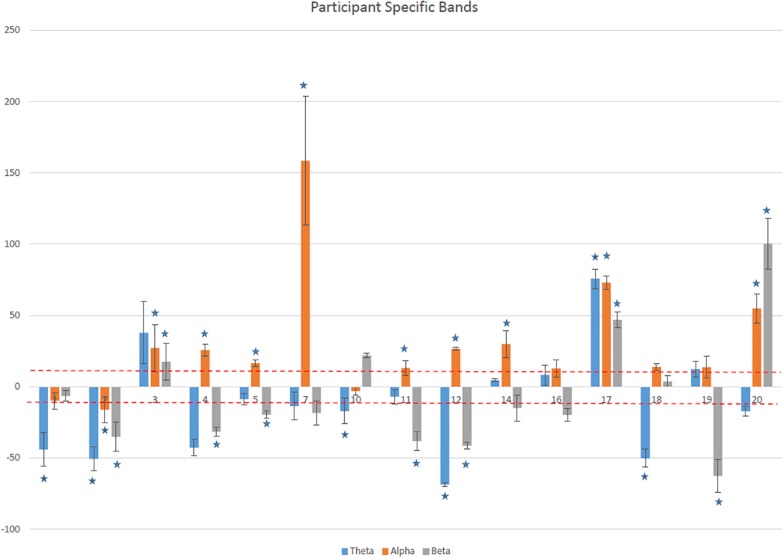
The average percentage of change (mean ± SD) over all training sessions for each participant in patient specific theta, alpha, and higher beta bands. Asterisks show statistically significant values.

Figure 4 shows the average (mean ± SD) modulation of power, with respect to the baseline power in participant specific frequency ranges for each participant. Twelve out of fifteen participants increased on average the alpha power for more than 10% and for nine of them this was statistically significant. Ten reduced their theta band power by more than 10% while in seven this downregulation was statistically significant. Six reduced their beta band.

There was a statistically significant difference in the alpha (*p* < 0.001) and theta band power (*p* < 0.04) calculated for fixed and participant specific ranges. For the beta bands this difference was not statistically significant (*p* < 0.08).

**FIGURE 5:**
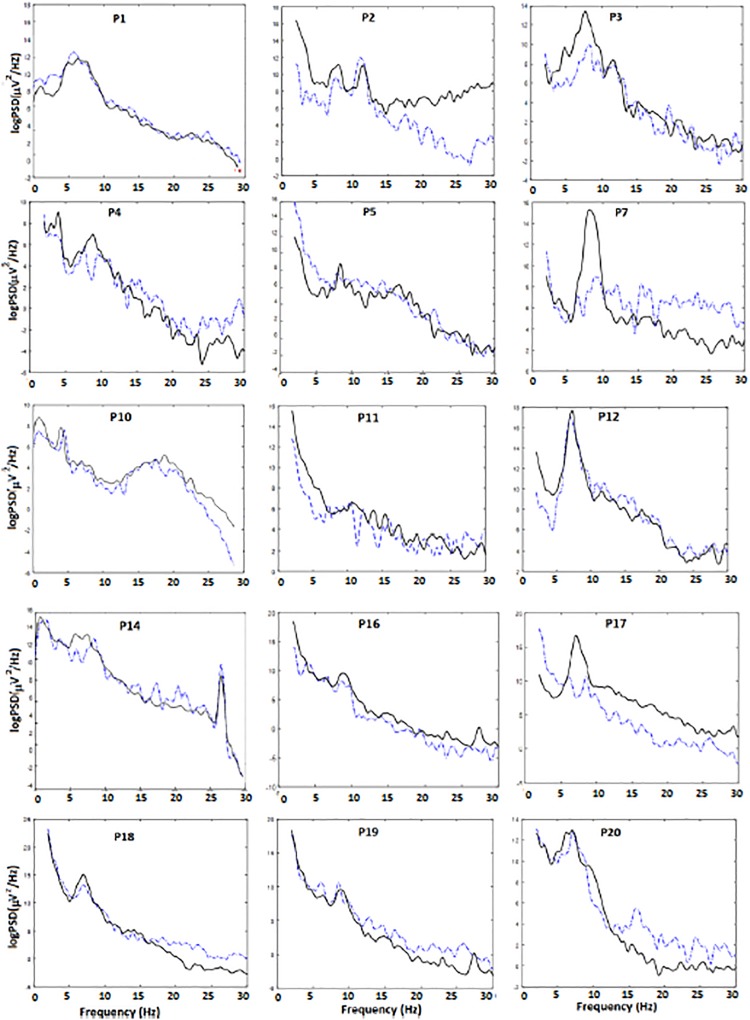
Power spectrum density during baseline (dashed line) and during NFB (solid line) for each single participant.

To further illustrate why it was necessary to calculate the alpha band power in a participant specific manner, we show a representative example of power spectrum density for each participant during the baseline period and during NFB in Figure 5. Participants average dominant alpha peak is shown in Table 3. All but one participant had a dominant alpha peak 8 Hz or lower, as opposed to the general population where it is about 10 Hz (Niedermeyer, 1999). The dominant peak did not change as a result of NFB.

**TABLE 3:** Individual dominant alpha peak.

**Participant**	**Alpha peak (Hz)**	**Alpha range with respect to the dominant peak (α ± 2) Hz**
1	8.0	6.0–10.0
2	6.5	4.5–8.5
3	8.0	6.0–10.0
4	8.0	6.0–10.0
5	8.0	6.0–10.0
7	8	6.0–10.0
10	6	4.0–8.0
11	6	4.0–8.0
12	8	6.0–10.0
14	8	6.0–10.0
16	8	6.0–10.0
17	9	7.0–11.0
18	7.5	5.5–9.5
19	7.5	5.5–9.5
20	8	6.0–8.0
Mean ± SD Median [Q1,Q3]	7.6 ± 0.9 8, [7.5,8]	

Figure 5 shows that during NFB participants did not shift their alpha peak in the 9–12 Hz range but increased the power in their own alpha range (see, e.g., P3, P4, P7, andP17). Six participants downregulated their beta band activity (desired direction) while four were increased their beta band activity (undesired direction). The individual theta band in most cases actually overlapped with the delta (1–4 Hz) band while individual alpha band partially overlapped with the theta (4–8 Hz) band. Furthermore in five participants (P5, P7, P11, P16, and P17) the dominant peak was barely visible in the relaxed state but emerged during NFB.

The alpha band power normally increases when a person closes their eyes. Engagement in a cognitive task normally results in decrease in alpha and increase in beta band activity (Niedermeyer, 1999). In addition to the wide spread alpha activity, the central cortex is also a source of the sensory motor rhythm, also present at 8–12 Hz (Niedermeyer, 1999). The idea behind NFB from electrode location C4 was to modulate the sensory-motor rhythm. This rhythm drops upon motor action, and there is no known verbalized strategy to increase it with the eyes opened. For this reason we believe that the increase in alpha (i.e., sensory motor rhythm) activity in this case is due to NFB and not due to general engagement in a cognitive task.

### The Effect of NFB on Pain

Table 4 shows the average level of pain reduction following NFB treatment for each participant. Because the VNS pain ratings are not normally distributed (e.g., the initial pain level in all participants was between 4 and 10) we present them as median and first and third quartile and related statistical results based on non-parametric statistical analysis. We, however, also present the same results as mean ± SD in the Appendix, similar to how results are presented in most pain related publications. Both approaches provide similar results.

**TABLE 4:** Pain intensity before and after NF training (median, first and third quartile Q1, Q3).

**No.**	**Pain Intensity**			***p*-value**
	**PreNF (VNS) Median, (Q1, Q3)**	**PostNF (VNS) Median, (Q1,Q3)**	**Change in Pain Intensity (%) with respect to Median**	
P1	9, (8,9)	6, (5, 7)	−33%	0.0001
P2	8, (8,9)	5.5, (5, 6)	−31%	0.0002
P3	9, (8,9)	5.5, (5, 6.25)	−38.9%	0.0022
P4	3, (3,3)	2.5, (2, 3)	−16.7%	0.0001
P5	5, (5,6)	4.5, (4, 5)	−10%	0.0001
P6	\	\	\	\
P7	4.5, (4,5)	3.5, (3, 4)	−20%	0.0449
P8	\	\	\	\
P9	\	\	\	\
P10	8, (8,9.75)	5.5, (5, 6)	−31%	0.0002
P11	4, (3.5,4.5)	3, (3, 3.5)	−25%	0.1667
P12	7, (7,8)	4, (4, 5)	−42.9%	0.0002
P13	\	\	\	\
P14	5, (5,6)	4, (3, 4)	−20%	0.0122
P15	\	\	\	\
P16	3, (2,3)	1, (0,1)	−66.7%	0.0001
P17	6, (5,6)	3, (2, 4)	−50%	0.0001
P18	5, (4,6)	4, (4, 5)	−20%	0.0967
P19	5, (5,7)	2.5, (1.75, 3)	−50%	0.0001
P20	7.5, (6.75,8)	6.5, (5, 7)	−13%	0.0649

*VNS, Visual Numerical Scale (VNS, 0 = no pain, 10 = worst pain imaginable); PreNF, before each NF session; PostNF, after NF session. Q1 and Q3 are 1st and 3rd quartiles.*

Twelve out of fifteen participants achieved statistically significant reduction in pain. In eight participants this was also clinically significant (larger than 30%). Thirty percent was based on studies reporting on the effectiveness of pharmacological treatments (Wiffen et al., 2017). If we adopt 15% as a clinically significant value, as in papers reporting on benefit of neurostimulation technology (O’Connell et al., 2018), thirteen participants achieved a clinically significant reduction in pain.

There was no direct correlation between the increase in alpha band power during NFB and decrease in pain (*p* = 0.4589, *R* = −0.0152). This is, however, not surprising bearing in mind that the NFB tunes the brain toward a homeostatic set point, that might not be a state with a maximum alpha band power. Baseline preNFB alpha power varies due to the circadian rhythm and was not possible to assess because participants did not necessarily always practice NFB at the same time of the day.

**FIGURE 6:**
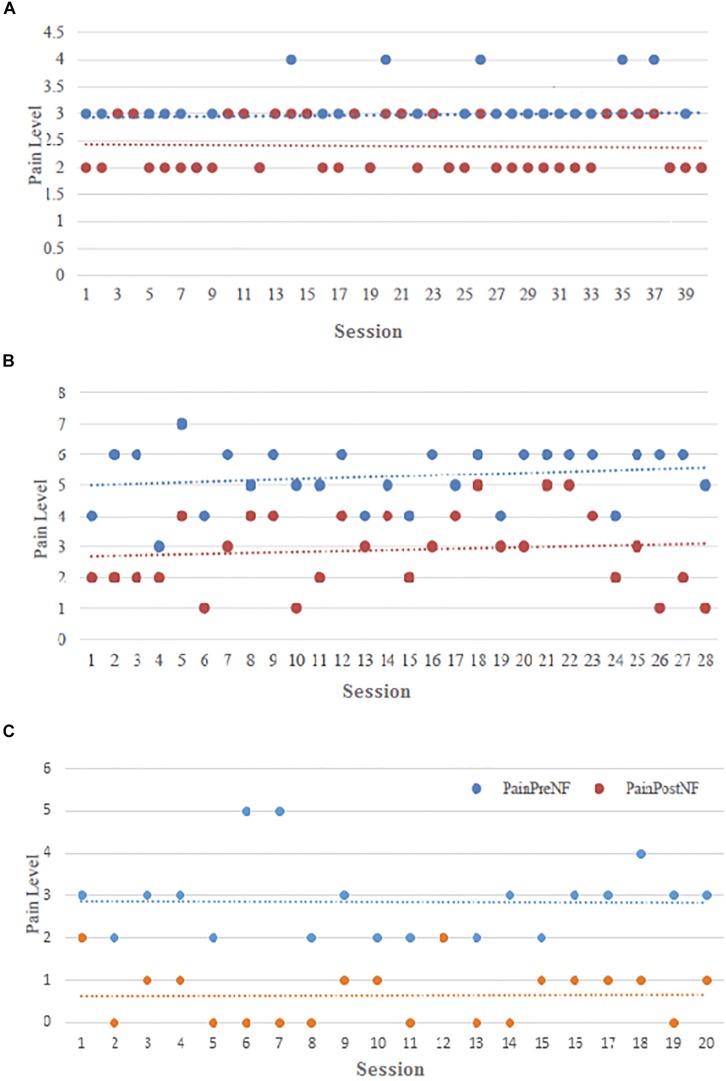
Pain intensity (Visual Numerical Scale) before (blue dots) and after (red dots) NFB over all training sessions for representative participants **(A)** P4, **(B)** P17, and **(C)** P16.

We analyzed how the baseline pain intensity changes over time. There was not a clear trend of Pre NFB pain level decrease, though in some patients relief of pain during NFB was almost compete (Figures 6B,C). This could be attributed to a fact that participants did not practice NFB at regular intervals but only when their pain level increased to the point that they needed pain relief. With that respect some participants consistently practiced NFB when their pain would reach the same level and achied about the same pain relief (Figure 6A) while in the others the pre and post NFB pain level varied, frequently reaching very low level (Figures 6B,C).

### A Relationship Between the Modulation of EEG Activity and the Intensity of Pain

We analyzed the relationship between a significant reduction in pain and a significant upregulation of the alpha power. There were four possible scenarios

(a) Participants significantly increased their individual alpha power and achieved a statistically significant reduction in pain.
(b) Participants significantly increased their alpha power but did not achieve a statistically significant reduction in pain.
(c) Participants did not significantly increase their alpha power but did achieve a statistically significant reduction in pain.
(d) Participants did not significantly increase their alpha power and did not achieve a statistically significant reduction in pain.

We showed that eight participants who significantly increased their alpha power also achieved a significant reduction in pain (P3, P4, P5, P7, P10, P12, P14, and P17), while four who did not achieve a significant increase in alpha power also achieved a significant reduction of pain (P1, P2, P16, and P19). However, three of these four had a significant reduction in beta or both theta and beta band power.

Furthermore two participants who achieved a significant increase in the alpha band power did not achieve a significant reduction of pain. One of these (P11) achieved a significant reduction in the beta band power while the other (P20) achieved a significant increase in beta power. Finally one participant who did not significantly increase the alpha band power did not significantly reduce pain (P18), this participant significantly reduced the theta band power.

### Relationship Between Pain and Demographic Data

Our analysis confirmed results from the literature regarding the weak relationship between the intensity of pain and the participants’ demographic data (Siddall et al., 2003). Spearman non- parametric rank sum test was applied. There was no significant correlation between the level of pain and the level of injury (*p* = 0.6949, *r* = 0.0935). The level of pain was not significantly correlated with the time since injury though the *p*-value was close to the significance level (*p* = 0.0631, *r* = 0.4231) indicating that pain might get worse over time. No significant correlation was found between the level of pain and the reduction of pain on the VNS (*p* = 0.81, *r* = 0.65), the dominant baseline power alpha frequency and the reduction in pain during NFB (*p* = 0.9703, *r* = −0.0105), the initial level of pain and the dominant alpha frequency (*p* = 0.4522, *r* = −0.2101), and time since injury and the reduction in pain during NFB (*p* = 0.9701, *r* = 0.1010). Likewise, there was no significant difference in the initial level of pain between walkers (ASIA D) and non-walkers (ASIA A and B) (*p* = 0.7528), nor between participants with incomplete (ASIA B, C, and D) and complete (ASIA A) injury (*p* = 0.6242).

There was no clear correlation between the duration of the study in weeks and the reduction of pain (*p* = 0.796, *R* = 0.094) and the total number of NFB sessions and reduction of pain (*p* = 0.299, *R* = 0.287). This may indicate that NFB may contribute to reduction in pain to a certain level, but that further implementation may only keep this pain level. It also has to be acknowledged that not every NFB session is successful as it depends on the participant’s mental state (Demos, 2005).

### Incidental Findings and Side Effects

Similar to our previous study, most participants reported a tingling sensations staring in their toes, or in case of tetraplegics also at the tip of their fingers. One participant that initially had CNP in the form of an excessive cold sensation, reported a sensation of pleasant warmth during NFB; the sensation of a pleasant warmth was also reported by the majority of participants whose pain was experienced as a burning sensation. Three participants reported successful transfer learning, i.e., applying NFB mental strategy without a device (it is possible that more participants had this ability but we did not systematically explored it). One participant said that wearing an audio headset at work produced similar sensation on his head which additionally helped him to imagine doing NFB. Transfer learning is an inherent property of successful NFB independent on a specific application (Sherlin et al., 2011) that might affected regularity of NFB practice with the device. It may be considered as the main advantage of NFB over tDCS or rTCS because it allows a person to apply a NFB strategy even without a device. Experience from other NFB studies (Sherlin et al., 2011), however, show that using NFB device at least once a week is necessary in order to keep the transfer learning ability.

Two participants with limited walking ability due to clonic spasm were spasm free immediately after 20–30 min of training, demonstrated in our laboratory. The spasm subsided for the rest of the day following NFB. Another participants who could walk with poor prioprioception reported returned sensation in his foot sole to the extent that he could feel whether the surface on which he walked was slippery or rough. He also demonstrated that, due to improved proprioception, he could stand still with his eyes closed, that is a challenging task for people with poor proprioception. Participants also occasionally mentioned improved sleep but it was hard to determine whether it was primarily a better nights sleep or better sleep quality due to reduced pain.

Negative side effects were occasional headaches, but it was unclear whether this was due to increased concentration or due to the NFB protocol. Another negative side effect was occasional hypersensitivity in the soles of the feet in the participant who regained proprioception, this was managed by reducing the duration of NFB.

We did not systematically examine participants’ mental strategy but on their check-up visit we asked what were they thinking of during NFB. They all mentioned evoking some happy episodic memory, such as thinking of a favorite activity, an episode with a favorite family member or a favorite holiday (e.g., imaging laying on a beach). This might be an indication that they activated default mode network (Sestieri et al., 2011).

## Discussion

With the advent of wearable consumer grade wearable EEG headsets, this technology become available to individual users. Thus neurofeedback becomes competitive with direct current stimulation technique, which has also been used for pain reduction, and can be purchased over the Internet. This prompted us to organize this feasibility participant self-managed NFB study. We demonstrate that after up to 4 training sessions at hospital, people naïve to EEG technology could learn how to use NFB on their own at home, reporting reduction in pain.

The most significant result of this study is the evidence that NFB is based on upregulation of participant specific rather than fixed frequency bands, which are lower than in healthy individuals. We show PSD modulation for each single participant, advancing our understanding of variation in neuromodulation strategies. Our previous study in a controlled hospital environment indicated a relationship between NFB and changes in pain related areas of the cortex (Hassan et al., 2015; Hasan et al., 2016). The NFB protocol, however, had three parameters and the number of participants was too small to establish which of these are most relevant for the reduction in pain. In this study we showed that nine out of fifteen participants achieved a significant modulation of their individual alpha band and that eight of those who achieved significant modulation of the alpha band also achieved a statistically significant reduction in pain. A study by Sarnthein et al. (2006) showed that patients who underwent central lateral thalamoctomy experienced immediate relief of CNP while their alpha peak frequency remained low 3 months post-surgery. Only 12 month post-surgery the frequency of the dominant alpha peak shifted toward higher frequencies (Sarnthein et al., 2006). Schulman et al. (2005) also showed that the dominant alpha peak shifted toward higher frequencies (energy in 9–11 Hz band increased) only in patients who reported 50% reduction of pain as a result of spinal cord stimulation. They did not report how long patients were pain free. Our study lasted on average under 3 months, so it is possible that prolonged NFB practice would result in alpha peak shift, under assumption that NFB would cause long term top down thalamic depolarization.

Although theta and beta band have clear relation to thalamo-cortical dysrhythmia, their relation to the reduction in pain through NFB was less clear than for the alpha band. One of the reasons might be that the bar representing the alpha power was placed in the middle of the GUI and was largest so participants might have perceived it as the most relevant. The other reason might be that the theta and beta bands overlap with eye movement artifact and muscular activity frequency range and could be reduced by minimizing these artifacts. A commercially available NFB software (e.g., NeXus, MindMedia) typically observe one frequency band and monitors the theta and higher beta band as a means of reducing eye movement and muscular artifacts rather than a proper NFB training. They typically present the central (e.g., 8–12 Hz, 12–15 Hz) band with the largest bar as it reliably presents EEG activity without the artifacts and is most relevant for the NFB protocol. On the other hand, minimizing the theta and beta activity also minimized the eye movement (theta) and muscular artifacts (beta).

We showed that 8 out of 15 participants achieved a clinically significant (larger than 30%) reduction in pain. We did not specifically check for how long the pain subsided, but judging by the frequency of use, the effect lasted from 1 to 3 days. This is comparable to the effect of medication and of other neurostimulation techniques (O’Connell et al., 2018). CNP is a chronic condition, and although NFB has a potential to target EEG features and to presumably restore the default mode network, the cause of CNP is the injury of the spinal cord which cannot be treated.

The main signatures of CNP are increased theta activity and reduced dominant alpha frequency (Sarnthein et al., 2006; Boord et al., 2008; Vuckovic et al., 2014). Reduced alpha activity has also been related to chronic pain in general (Camfferman et al., 2017). More recently we identified reduced alpha activity and reduced dominant alpha frequency as early, predictive markers of CNP (Vuckovic et al., 2018b). In the current study we show that individual alpha band in most participants has shifted toward the theta band frequency range. We also showed that none of the participants who reported reduced pain had increased their individual alpha peaks, which stayed around 8 Hz. From this we may conclude that the level of alpha synchronization, rather than its peak frequency is related to the cause of pain. Reduced dominant frequency has been also reported in people with SCI who do not suffer from CNP (Tran et al., 2004). Increased alpha power in people with SCI related CNP, reported in some previous studies (Jensen et al., 2013b) may be at least partially related to the fact that researchers were looking in too high frequency range.

Peak alpha band frequency varies among healthy individuals and is age dependent (Niedermeyer, 1999). From that reason, some NFB studies are based on individual frequency bands with respect to the individual peak alpha frequency α_p_ (Quaedflieg et al., 2016; Lavy et al., 2019).

Increasing the alpha power, effectively means increasing the level of synchronization, i.e., bringing the sensory-motor area to an inactive state. Several EEG and fMRI studies indicated that CNP in the SCI population is related to the overactive sensory-motor cortex (Sarnthein et al., 2006; Gustin et al., 2010) and we also showed that prolonged NFB practice relates to decreased event related desynchronization during imagined movements of both painful and non-painful limbs (Hasan et al., 2016).

There are several limitations of this study. First of all we did not have a control group, as this would have been difficult and potentially unethical to organize in a self-managed study taking place over several months and requiring lots of participants’ engagement.

The role of placebo in pain treatments should not be underestimated (Cragg et al., 2016), as even the randomized controlled trial with one of the most widely used medication for CNP, pregabalin, showed that while about half of the participants taking gabapentin experienced a moderate reduction in pain (30%) and at the same time, one third of people taking placebo also experienced a moderate reduction in pain (Wiffen et al., 2017). The placebo effect might be even higher in NFB due to the fact that participants were given active “control” over their pain. We could only show that 2/3 of people who actively regulated their alpha brain waves also achieved a significant reduction in pain. On the other hand, due to “transfer learning,” i.e., the ability to reduce pain by brining themselves into “neurofeedback” state even without using the device it would be hard to test for placebo in people who are experienced in NFB.

Although NFB is a popular technique for the treatment of various neurological problems, there are few published pain related randomized controlled NFB studies. A randomized controlled studies for the treatment of fibromyalgia, suggested a protocol that upregulated the 12–15 Hz band and downregulated the theta and higher beta (22–30 Hz) band from Cz (Caro and Winter, 2011). Similar to our study they selected the motor cortex and had a most relevant frequency band to upregulate and two additional to downregulate. They reported a reduction in pain of 39% but did not provide any EEG evidence that participants successfully modulated their brain waves. Kayiran et al. (2010) performed a randomized control trial to compare the efficacy of NFB with the efficacy of medication for the treatment of fibromyalgia, using a similar protocol as Caro and Winter (2011). They claimed that NFB that increases a 12–15 Hz band power appears to facilitate a thalamic inhibitory mechanisms. They reported that the NFB group achieved larger benefits than the control group in terms of reduction in pain and other parameters related to sleep, depression and quality of life. They provided results averaged over all 18 participants about changes in the theta, alpha and beta power over different weeks. The only group who looked at the effectiveness of NFB for the treatment of CNP tested three different protocols (none being the same as ours) over four NFB sessions each and found modest, clinically non-significant reduction in pain (Jensen et al., 2013a). The protocols were based on increasing alpha or 10–15 Hz while decreasing beta or theta and beta bands from several different locations. They reported that this effect persisted at 3 months follow up. However, they later suggested that NFB may be a useful technique to enhance the effect of hypnotic analgesia (Jensen et al., 2016).

Motor imagery (MI) is a self-induced neuromodulation technique based on a verbalized strategy used in SCI both as a therapy and a method to reduce CNP (Moseley, 2007). Similarity between MI and our NFB protocol is that both modulate the alpha/sensory-motor rhythm. MI results in the suppression of SMR (movement event related desynchronisation) (Pfurtscheller and Lopes da Silva, 1999) while our protocol rewards the increase of SMR (synchronization). Gustin et al. (2010) showed that prolonged MI in people with CNP due to SCI increases pain. With that respect, one might expect that the opposite, i.e., increase of SMR would reduce CNP as shown in our study.

On the contrary, Moseley (2007) showed that motor imagery combined with a visual illusion reduces neuropathic pain in people with SCI. However, Moseley recruited mostly people with at level pain while in Gustin et al. (2010) participants had pain had under the level of injury CNP. In a case report, Yoshida et al. (2016) also showed that motor imagery training to increase SRM resulted in reduced neuropathic pain in a single participant with SCI who received 4 months of training. A systematic review of application of MI in SCI shows that MI has rehabilitation benefits but that effect on pain is inconclusive (Aikat and Dua, 2016).

The other limitation of our study is that the total duration and the total number of NFB sessions varied among participants. However, as one of the primary goals was to observe the self-managed usage pattern, this provided us with valuable information about frequency of use of NFB which for most participants was about three times a week for 20–30 min.

In this study most participants were taking drugs, such as antidepressants or anticonvulsants, which in large doses (more than normally prescribed) might increase the frequency content in the theta band (Bauer and Bauer, 2005; Wauquier, 2005). To the best of our knowledge all participants received medications in doses prescribed by a medical specialist.

Finally because it was necessary to reduce the setup time, EEG was recorded from one electrode only, which was sufficient to understand the neuromodulation strategy but did not allow for the exploration of the effect of NFB on wider cortical structures.

A randomized controlled trial with a modified NFB protocol, focused on participant individual alpha band should be performed to confirm the efficiency of the therapy. The analysis of non-oscillatory, scale-free cortical activity (e.g., fractals, long range temporal correlations (Dimitriadis and Linden, 2016; Kesiæ and Spasiæ, 2016) should confirm whether NFB bring the neural system in the state of self-organizing criticality Hernandez-Urbina and Michael Herrmann, 2016) which is considered a state of homeostatic set-point (Ros et al., 2014). A study in controlled conditions would also provide an opportunity to regularly test the mental states at the time of NFB (STAI form Y1-Y2 (Spielberger et al., 1983), BDI-II (Beck et al., 1996) and workload (NTLI NASA, 1986) as it might affect both participants ability to modulate their brain waves and to perceive the benefit of the therapy.

In this study all participants used the same protocol and the same electrode location, irrespective of the location of pain. In our previous study (Hassan et al., 2015) we showed that C3 was also effective in reducing pain but that it occasionally evoked spasms. A Cochrane review of rTMS and rDCS studies also showed that studies that targeted the motor cortex do not necessarily stimulate in a somatotopic manner with respect to the location of perceived pain. We believe taht the underlying mechanism is common in rTMS, tDCS, and NFB. CNP is seen by some researchers as a disrupted homeostatic state, in particular disrupted thermosensory inhibitory process (Craig, 2002). Thus neuromodulatory techniques for treatment of CNP maybe seen as therapies that tune the cortical activity toward a homeostatic setpoint. The main advantages of NFB over the other two modalities are that it potentially has less side effects as it does not apply an external electrical or magnetic stimulation, does not require an additional device and most importantly provides to participants an external locus of control and empowers them to actively contribute toward the reduction in pain. Hypnosis is another neuromodulatory technique which is believed to have a similar neuromodulatory mechanism to NFB, rTMS, and tDCS (Jensen et al., 2016).

Neurofeedback practitioners often set a variable NFB threshold which self-adjusts based on the last several minutes of NFB with the intention of adjusting the difficulty level for the participant’s performance. We used a fixed threshold in this study as it would have been difficult to perform quantitative analysis based on a variable threshold and also because variable threshold could lead to training in the opposite direction of the desired one (Ros et al., 2014).

Self-reported neuromodulation techniques through NFB included some pleasant episodic memories which indicate possible involvement of the default mode network (Sestieri et al., 2011). Our collaborators who used the same NFB equipment and protocol as our group tested the NFB mental strategy in ten participants with SCI and CNP (Anil et al., 2019). They showed that a mental state (e.g., attentiveness) rather than a mental strategy (e.g., imagination), was associated with neuromodulation success. They also showed that people who were unsuccessful in self-regulating their brainwaves also could not differentiate between successful and unsuccessful mental strategies.

In conclusion NFB may provide a clinically significant reduction of pain that lasts up to several days and people experienced with the technique may apply it even without the feedback. During NBF which results in reduction of CNP, people modulate their specific rather than fixed frequency bands. People with CNP can use NFB on their own without help of a trained professional. NFB does not provide cure for CNP and similar to pharmacological treatments would require long term use.

## Data Availability

The datasets generated for this study are available on request to the corresponding author.

## Author Contributions

AV designed the study, contributed to the data collection and analysis, and wrote the manuscript. MKHA collected and analyzed the data. MF and MP selected the participants and contributed to the data interpretation, and wrote the manuscript. CM contributed to the data analysis and wrote the manuscript.

## Conflict of Interest Statement

The authors declare that the research was conducted in the absence of any commercial or financial relationships that could be construed as a potential conflict of interest.

## APPENDIX

**TABLE A1:** Average pain intensity of each single participants before and after NFB training.

	**Pain Intensity**			
**No.**	**PreNF (VAS) mean ± SD**	**PostNF (VAS) mean ± SD**	**Change in Pain Intensity (%)**	***p*-value**
1	8.7 ± 1.2	6.0 ± 1.0	**−31%**	**0.007**
2	8.5 ± 0.7	5.5 ± 0.7	**−35%**	**0.001**
3	8.7 ± 0.6	5.3 ± 0.6	**−38%**	**0.002**
4	3.0 ± 0.5	2.4 ± 0.5	−18%	**0.5e^–5^**
5	5.1 ± 0.6	4.5 ± 0.6	−10%	**0.5e^–6^**
6	–	–	–	–
7	4.5 ± 0.9	3.5 ± 0.9	–22%	**0.001**
8	–	–	–	–
9	–	–	–	–
10	9.0 ± 0.7	5.5 ± 1.8	**−38%**	**0.04**
11	4.0 ± 1.0	3.3 ± 0.6	−15%	0.08
12	7.25 ± 0.5	4.27 ± 0.5	**−34%**	**0.002**
13	–	–	–	–
14	5.3 ± 1.0	3.7 ± 0.8	−28%	**0.003**
15	–	–	–	–
16	2.85	0.65 ± 1.3	**75%**	**0.5e^–5^**
17	5.4 ± 1.0	2.9 ± 1.3	**−46%**	**0.5e^–11^**
18	5.3 ± 2.1	4.7 ± 2.3	−15%	0.09
19	5.8 ± 1.7	2.3 ± 1.7	**−65%**	**0.0006**
20	7.3 ± 0.6	6.7 ± 0.6	−9%	0.09

*VAS: Visual Analogue Scale (VAS, 0 = no pain, 10 = worst pain imaginable); PreNF: before each NF session; PostNF: after NF session.*
